# Time lagged information theoretic approaches to the reverse engineering of gene regulatory networks

**DOI:** 10.1186/1471-2105-11-S6-S19

**Published:** 2010-10-07

**Authors:** Vijender Chaitankar, Preetam Ghosh, Edward J Perkins, Ping Gong, Chaoyang Zhang

**Affiliations:** 1 School of Computing, The University of Southern Mississippi, MS 39402, USA; 2 Environmental Laboratory, U.S. Army Engineer Research and Development Center, 3909 Halls Ferry Road, Vicksburg, MS 39180, USA; 3 SpecPro Inc., 3909 Halls Ferry Road, Vicksburg, MS 39180, USA

## Abstract

**Background:**

A number of models and algorithms have been proposed in the past for gene regulatory network (GRN) inference; however, none of them address the effects of the size of time-series microarray expression data in terms of the number of time-points. In this paper, we study this problem by analyzing the behaviour of three algorithms based on information theory and dynamic Bayesian network (DBN) models. These algorithms were implemented on different sizes of data generated by synthetic networks. Experiments show that the inference accuracy of these algorithms reaches a saturation point after a specific data size brought about by a saturation in the pair-wise mutual information (MI) metric; hence there is a theoretical limit on the inference accuracy of information theory based schemes that depends on the number of time points of micro-array data used to infer GRNs. This illustrates the fact that MI might not be the best metric to use for GRN inference algorithms. To circumvent the limitations of the MI metric, we introduce a new method of computing time lags between any pair of genes and present the pair-wise time lagged Mutual Information (TLMI) and time lagged Conditional Mutual Information (TLCMI) metrics. Next we use these new metrics to propose novel GRN inference schemes which provides higher inference accuracy based on the precision and recall parameters.

**Results:**

It was observed that beyond a certain number of time-points (i.e., a specific size) of micro-array data, the performance of the algorithms measured in terms of the recall-to-precision ratio saturated due to the saturation in the calculated pair-wise MI metric with increasing data size. The proposed algorithms were compared to existing approaches on four different biological networks. The resulting networks were evaluated based on the benchmark precision and recall metrics and the results favour our approach.

**Conclusions:**

To alleviate the effects of data size on information theory based GRN inference algorithms, novel time lag based information theoretic approaches to infer gene regulatory networks have been proposed. The results show that the time lags of regulatory effects between any pair of genes play an important role in GRN inference schemes.

## Background

A GRN is a complex set of highly interconnected processes that govern the rate at which different genes in a cell are expressed in time, space, and amplitude. Such a network is commonly represented by many pairs of proteins and genes, in which one protein/gene regulates the abundance and/or activity of another protein/gene [1]. GRN’s can be modelled and simulated using various mathematical and computational approaches [2]. The modelling and simulation of GRN’s is performed over the cDNA microarray data. There are two types of DNA microarray data: time series and time independent (or steady state). The time series data are obtained by sampling temporally the measurement process, whereas time-independent data sets are obtained by recording the gene expressions from independent sources, for example, different individuals, tissues, and experiments [3]. As time series data would enable one to capture the time varying nature of a GRN, it is the preferred form of data used in reverse engineering algorithms. Moreover, time series data only gives the expression levels of genes without any knowledge of other cellular elements like protein/metabolite concentrations. In this paper a GRN is represented as a graph which consists of a set of nodes that represent genes and a set of edges that represent the interactions between genes. Thus the GRN inference problem investigated in this paper refers to finding the regulatory relationship between the genes of an organism.

Reverse engineering of gene regulatory networks remains a major issue and area of interest in the field of bioinformatics and systems biology. A survey paper [4] discusses a number of models related to this area, viz. Bayesian Networks [6], Dynamic Bayesian Networks [7], Boolean Networks [8], Probabilistic Boolean Networks [9,10], Differential Equation Models [11] and Information Theory Models [3,12-15]. There is no gold standard method to reverse engineer gene regulatory networks; each method has its own advantages and disadvantages. Based on simulations of different models, it has been observed that differential equation models and dynamic Bayesian networks provide higher accuracy, but they are computationally expensive and hence, are applicable for only a small data set. Boolean networks can be used to study the *coarse grained* properties of genetic networks [9]. Such binary representation of gene expression is clearly an approximation, as most biological phenomena manifest their properties in the continuous domain.  Even though it is inherently deterministic, the Boolean formalism has enjoyed success in predicting biological behaviour, such as the accurate qualitative distinction between known tumor sub-classes [9,10].  This work suggests that meaningful biological information is not lost when measured, continuous-domain, gene expression data is made binary. Information Theoretic methods to reverse-engineer GRNs build on such Boolean network models of gene expression and have gained popularity due to their simplicity and less computational cost [4]. Each of the information theoretic schemes discussed in this paper as well as DBNs however can be easily extended to handle multiple levels of quantization to achieve higher inference accuracy at the cost of computational overhead.

ARACNE [14] and REVEAL [15] are two popular Information Theoretic approaches towards GRN inference. Both of these methods establish relationships between genes based on the MI metric. Zhao [3] analyzed the limitations of MI and proposed the conditional mutual information (CMI) based approach to infer GRNs. One of the major disadvantages of Information Theoretic approaches is the selection of the MI and CMI thresholds, for which Zhao [12] proposed the Minimum Description Length (MDL) principle and showed its effectiveness in selecting the best MI threshold. The MDL principle states that if multiple theories exist, the one with the minimum description length is the optimal. However the definition of description length varies for different models and applications. In their MDL implementation, Zhao [12] defined the description length as the sum of the model length (expressed as the memory usage of the inference algorithm) and data length (expressed as the over-all entropy of the inferred network). One limitation of the MDL principle was that the model length quantity in the description length expression could make the implementation arbitrary [16]. To circumvent this problem, we have earlier proposed the Predictive Minimum Description Length (PMDL) principle approach [17] wherein we showed that by removing the model length quantity from description length and using CMI a higher inference accuracy can be obtained. 

MI and CMI metrics are central in establishing the relationships between genes in information theory models. Hence, in order to design a smart GRN inference algorithm, it is important to study the behaviour of these MI and CMI metrics on microarray data of various sizes. The MDL implementation of Zhao [12] will henceforth be referred to as “network MDL” in the rest of the paper.

Another major disadvantage in information theory based models is that MI and CMI do not give directions between relationships. A unit time delay was assumed in our earlier PMDL [17] implementation. Zou [18] showed that the time lags in regulating one gene by another play an important role in inference accuracy as evident in their Dynamic Bayesian Network based approach. To incorporate the effects of time-lags in information theoretic methods, we propose a new time lag computation method in this paper, which is used to modify the standard MI and CMI computations. Based on our modified MI and CMI metrics, we next present novel time-lagged GRN inference schemes that show promising results in terms of improving the inference accuracy.

Our major contributions in this paper can be summarized as follows:

1. We show that the performance of the inference algorithms saturate beyond a certain data size due to  the saturation in the information theory metric mutual information. Note that we have only varied the data size in our experiments to understand the effects of regulatory time-lags between genes on the algorithms. The overall performance of the algorithms would also be affected by other factors (e.g., the number of replicates and number of external chemicals used in the experiments to name a few), which might lead to other novel innovations that need to be considered in designing reverse-engineering schemes. This is however outside the scope of this paper. 

2. A new way of computing time lags between any pair of genes is presented. Our scheme makes sure that time lags cannot be negative and we argue that a more biologically pragmatic view is that a gene can affect another gene only when it is up-regulated. This assumption makes more sense in the Boolean network formalism of GRNs where a gene can only be in two possible states: ON (i.e., up-regulated) and OFF (i.e., down-regulated).

3. We introduce the time lagged Mutual Information (TLMI) and time lagged Conditional Mutual Information (TLCMI) quantities.

4. We present novel GRN inference schemes based on TLMI, TLCMI, MDL and PMDL principles that provide higher accuracy over the existing information theoretic methods.

## Results

In this section, we first report the results of the existing inference schemes that were run on the time-series micro-array data of varying size and illustrate that the performance of the methods saturates beyond a certain number of time points. We also report how the pair-wise MI metric saturates beyond a certain data size. We then present our new time lag computation scheme and the modified version of the network MDL algorithm wherein, we replace the MI metric which considers unit time delay with the TLMI metric (considering a time-lag of τ). We next present a modified version of the PMDL algorithm, by replacing the MI and CMI metrics with the TLMI and TLCMI metrics. Finally the results from the network MDL, PMDL and modified network MDL and PMDL algorithms are compared.

### Parameters to evaluate inference accuracy

Benchmark measures recall *R* and precision *P* are used to evaluate the performance of the algorithms. Although different definitions for recall and precision exist in the literature [20], in this paper, *R* is defined as C_e_/(C_e_+M_e_) and *P* is defined as C_e_/(C_e_+F_e_), where C_e_ denotes the edges that exist in the true network and in the inferred network, M_e_ are the edges that exist in the true network but not in the inferred network, and F_e_  are edges that do not exist in the true network but do exist in the inferred network.

### Synthetic data generation methodology for the in silico experiments

The performance of information theory and DBN based algorithms over different data size was carried out over random synthetic networks which were generated by the Genenetweaver tool [21-23].

It was imperative for us to use synthetic data over time series micro-array experimental data in this phase due to the following reasons:

• Very few experimental data sets have equal time intervals between experiments and also the data size is generally limited to around 20 time points. In our *in silico* runs, we wanted to keep equal time intervals between each time point data such that we can understand the true effects of regulatory time-lags between genes on the inference accuracy. It is generally not possible to assign a single time-lag value to a gene-pair if the expression readings for each time point were not evenly spaced as mentioned in Zou [18]. 

• Also, the saturation in inference accuracy generally requires a larger data size (> 30 time points as shown later) and it would have been difficult to identify the role of MI in bringing about this theoretical limit on the accuracy of information theoretic schemes with a smaller biological data set (of ~20 time points).

• It should be noted that the Genenetweaver software derives the *in silico* GRNs from the prior knowledge database of yeast (*Saccharomyces cerevisiae)* which contains 4441 genes and 12873 interactions. Thus in order to create a sample GRN with 10 nodes, Genenetweaver clusters the yeast transcriptomic network into modules and chooses the module having number of genes closest to the given input (in this case 10 genes) to create the *in silico* network. Each such module maps to a particular biological function and this strategy essentially ensures that there is minimum cross-talk of these set of genes with the others in the yeast network resulting in a higher efficacy of the inference algorithms that use them.

### Biological network data generation methodology to evaluate performance of proposed algorithms

The time series DNA microarray data from Spellman et al [25] was used to infer gene regulatory networks using the proposed algorithms. The Spellman experiment was chosen because it provides a comprehensive series of gene expression datasets for the Yeast cell cycle. Four time series expression datasets were generated using four different cell synchronization methods: cdc15, cdc28, alpha-factor and elutriation with 24, 17, 18 and 14 time points respectively. The alpha-factor dataset contained more time points than cdc28 and Elutriation datasets with fewer missing values than cdc15. Therefore, we chose to use time series expression data from the alpha-factor method to infer the gene regulatory networks.

We used the same preprocessing steps as in [12]. Initially the data is quantized to 0 or 1. In order to quantize the expression values of every gene, they are sorted in ascending order and the first and last values of the sorted list are discarded as outliers; then the upper 50% is converted to 1 and the lower 50% is converted to 0. Any missing time points are set to the mean of their respective neighbors. If the missing time point is the first or the last one, it is set to the nearest time point value.

Four separate biological networks (as discussed later) used for comparison purposes were derived from the yeast cell cycle pathway [26-28]. The fine tuning parameter required by the network MDL based algorithms is set to 0.1 to retain most of the connections (see [12] for more details on this).

### Effects of data size on GRN inference

#### Effects on Information Theory models

**Figure 1:**
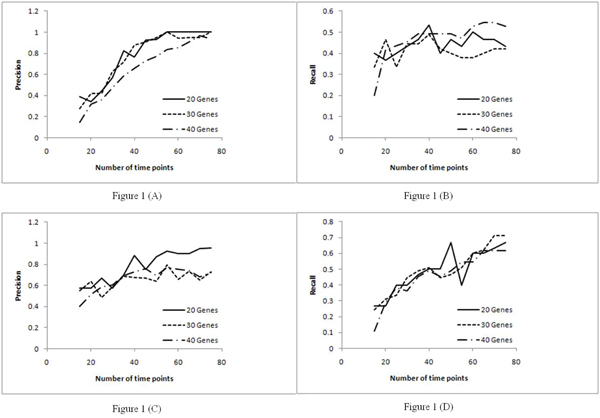
**Precision and recall curves for PMDL and network MDL over different data sizes.** Figure 1(a) – Precision curve for PMDL. Figure 1(b) – Recall curve for PMDL. Figure 1(c) – Precision curve for network MDL. Figure 1(d) – Recall curve for network MDL.

A network and data set with 75 time points was generated as in [12]. The resulting data set had expression levels quantized to two levels. Each of the algorithms was run 13 times starting with the first 15 time points. Increments of five time points were made for every subsequent run. And, for every run, the values of precision and recall were computed. In the network MDL algorithm, the free parameter was set to 0.2, and in the PMDL algorithm, the conditional mutual information threshold was set to 0.1 for best performance as reported in [17]. The plots for precision and recall are shown in Figure 1.

For the PMDL algorithm, it was observed that the precision increased until 55 time points, and, beyond that, the precision remains relatively stable for the two smaller tested networks (with 20 and 30 genes respectively). For the larger network (with 40 genes), the precision increased until 70 time points before saturation. The recall for PMDL algorithm increased until 40 time points before saturation for each of the 3 tested networks. For the network MDL algorithm, it was observed that precision increased until 35 time points and fluctuated after that. The recall for the network MDL algorithm kept increasing for all the test cases with considerable fluctuations.

**Figure 2:**
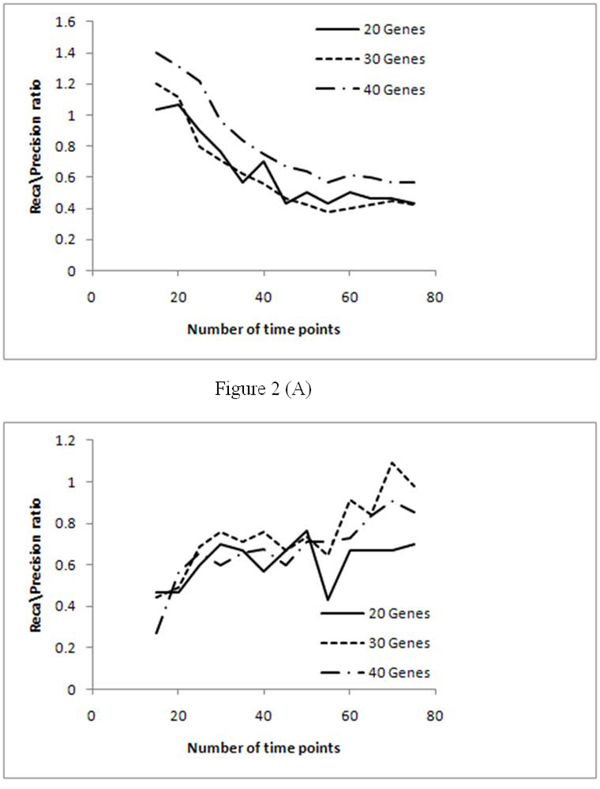
**Recall/precision ratio curves from PMDL and network MDL over different data sizes.** Figure 2(a) – Recall/precision ratio curve for PMDL. Figure 2(b) – Recall/precision ratio curve for PMDL.

For further analysis we considered the recall/precision ratio as shown in Figure 2. As seen in Figure 1, the recall and precision for the two information theoretic algorithms achieved saturation with increase in the number of time points. To approximately identify the minimum number of time points required to achieve maximum inference accuracy, we have used the recall/precision ratio metric. Hence, the number of time points where the inference accuracy achieves saturation will point to the approximate data size required to achieve best performance for each of these algorithms.

Table 1 summarizes the performance saturation points of the methods for 20, 30, and 40 gene networks. The saturation points for 20, 30, and 40 gene networks in PMDL were obtained at 55, 40, and 45 time points, respectively. In the case of the network MDL scheme, multiple saturation points were observed. For a 20 gene network, saturation was obtained at 30 and 60 time points. In the case of a 30 gene network saturation was obtained at 30 and 60 time points. Finally in a 40 gene network, performance saturation was obtained at 30, 45, and 70 time points. As description length of network MDL involves both data and model length, the performance of the method fluctuates considerably at various time points, which is not the case for PMDL, whose description length involves only the data length. Essentially, PMDL minimizes only the data length, and hence the entropy in the network structure (without considering the memory requirements measured by the model length in network MDL) thereby achieving steady saturation in performance. Hence, this analysis also points to the higher applicability of the PMDL scheme over network MDL as a data size beyond the saturation point will always guarantee the best performance, which is not the case for the network MDL based scheme.

**Table 1:** Performance saturation points

MethodNo. of Genes	PMDL	Network MDL
20	55	30, 60
30	40	30, 60
40	45	30, 45, 70

#### Effects on DBN based scheme

**Figure 3:**
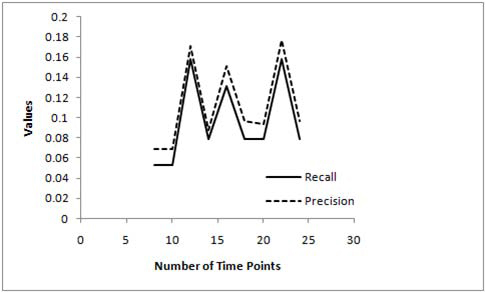
**Precision and recall curves for DBN based scheme**.

To understand the performance implications of the more conventional DBN approach on the number of time points, we conducted similar experiments with the DBN scheme developed by Zou [18]. As the time complexity of this DBN approach increased exponentially with the number of time points, we studied the effects on inference accuracy for a smaller dataset. We generated a 20 gene network with 24 time point’s data using the Genenetweaver tool [21-23].  Since the algorithm required a minimum of 8 time points, we studied the effects from 8 time points to 24 time points with steps of two as illustrated in Figure 3.

**Figure 4:**
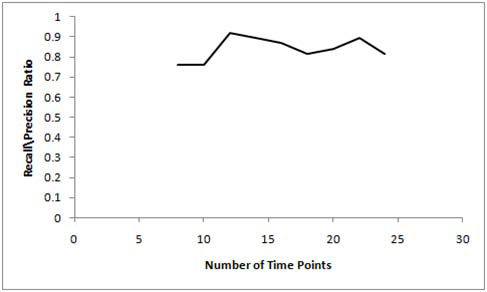
Recall/precision ratio curves for DBN based scheme.

From Figure 3 it is seen that neither the precision nor recall parameters for the DBN approach saturate as the number of time points are increased. In fact, the best precision is achieved for data sizes of 10 and 22 time points (higher the precision, better the accuracy), whereas, the best recall is achieved for data sizes of 8 and 10 time points (lower the recall, higher the accuracy). However, both precision and recall fluctuates appreciably with increase in the data size resulting in high fluctuations in the recall/precision ratio metric as well (Figure 4). These results somewhat non-intuitively suggest that the DBN approach achieves best performance for a lower data size (~10 time points) as the recall/precision ratio is the lowest for data sizes of 8-10 time points (from Figure 4). Thus the DBN approach does not necessarily achieve better performance as the data size is increased making it difficult for biologists to devise the right experiments. However, we need to conduct more comprehensive tests on the DBN approach (with different network sizes and more number of time points) before we can make this conclusion although the high time complexity of this approach makes it increasingly difficult to run the test cases.

### Why do information theory based models saturate?

**Figure 5:**
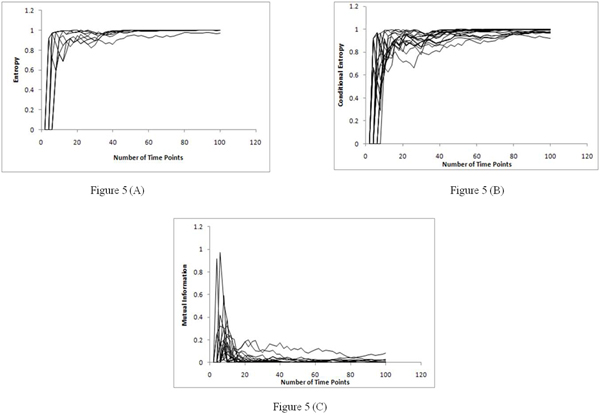
**Entropy, conditional entropy and mutual information curve over various data sizes.** Figure 5(a) – Entropy curve over various data sizes. Figure 5(b) – Conditional entropy curve over various data sizes. Figure 5(c) – Mutual information curve over various data sizes.

The performance saturation of the methods motivated us to study the behavior of the information theoretic quantities of entropy, conditional entropy and mutual information. For these set of experiments, biological synthetic data was created using the Genenetweaver tool [21-23]. We built a five gene network and produced synthetic data based on 100 time points. The synthetic data was quantized to two levels and then the information theoretic quantities were calculated.  Figure 5 shows the plots for entropy, conditional entropy and mutual information. We have computed the entropy of all the genes in the network across 100 time points while the conditional entropy and MI were computed for each pair of genes. We find that with more data (and correspondingly with more time points) both the entropy and conditional entropies increase in the network (tending to unity) resulting in very low values for MI (which tends to zero).

The plots conclude that the saturation in the methods was due to the saturation in the mutual information quantity which goes close to zero even though the entropy increases in the network. This would conceptually mean that there is room to improve on the inference accuracy (due to high entropy), yet the mutual information metric will not be able to point us to the right direction. Other information theoretic algorithms, like REVEAL [15] use the ratio of MI and entropy to infer the network for this purpose which supposedly gives good performance. However, from the entropy and mutual information curves in Figure 5, we can see that the ratio of mutual information and entropy will also saturate, as the entropy increases in the network, and hence this ratio might also not be the right metric to achieve better accuracy by making use of more time point’s data. The recently proposed Directed Mutual Information metric [24] might be a better metric than the conventional MI based algorithms. We do plan to conduct similar studies on the performance of GRN inference algorithms based on these different metrics as a function of the number of time points in the future. It is imperative to identify the right metric for the research community to decide which class of GRN inference algorithms can work best with time-series data and also understand the ideal data size for them. 

The saturation in MI due to increasing number of time points would suggest that the MI should not be computed for the entire range of time points of micro-array data available from the experiments. GRNs are inherently time varying, and hence the pair-wise MI between any 2 genes needs to be computed over the time range where the first gene will have *substantial regulatory effect* on the other one. This can be best approximated by estimating the regulatory time-lags between each gene pair, and subsequently computing the MI between them for this particular time range. This concept was used to compute the time-lags between genes and the TLMI and TLCMI metrics as discussed in the Methods section. Note that, the time lag computation concept initially proposed in [18] to implement time-lagged DBN needs to change to avoid the case of negative time-lags.

### TLMI based network MDL implementation

**Figure 6:**
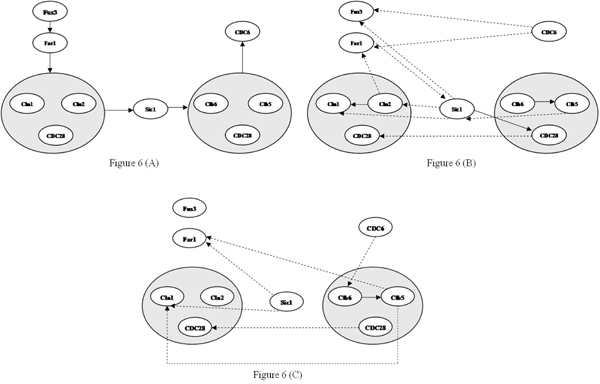
**First biological network inference results using TLMI based network MDL and MI based network MDL.** Figure 6(a) – True network. Figure 6(b) – Network Inferred using TLMI based network MDL. Figure 6(c) – Network Inferred using MI based network MDL.

A network with 10 genes was derived from the yeast cell cycle [26-28] and Spellman’s data [25] was used for the gene expression values at different time-points. The TLMI implementation of the MDL algorithm inferred 12 edges of which three were correct where as MI implementation [12] inferred seven edges of which one was correct. These initial results favor our approach. Figure 6 shows the true network and networks inferred using TLMI based network MDL and network MDL algorithms.

**Figure 7:**
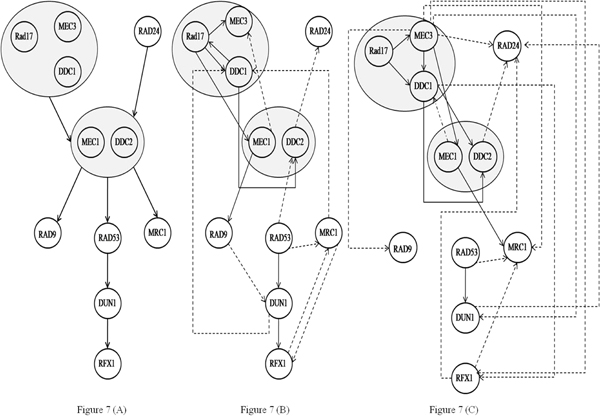
**Second biological network inference results using TLMI based network MDL and MI based network MDL.** Figure 7(a) – True network. Figure 7(b) – Network Inferred using TLMI based network MDL. Figure 7(c) – Network Inferred using MI based network MDL.

**Figure 8:**
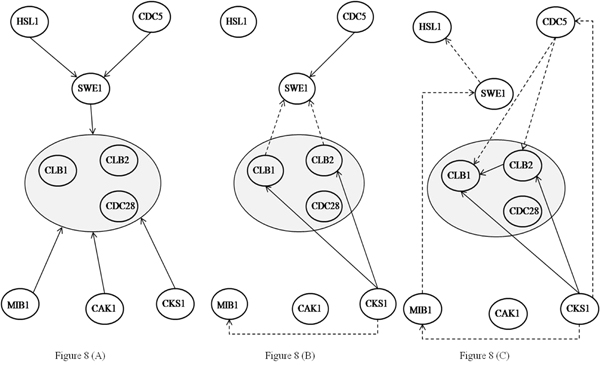
**Third biological network inference results using TLMI based network MDL and MI based network MDL.** Figure 8(a) – True network. Figure 8(b) – Network Inferred using TLMI based network MDL. Figure 8(c) – Network Inferred using MI based network MDL.

We repeated the same process for two other biological networks with 11 and nine genes from the yeast cell cycle and Spellman’s data [25]. The corresponding results are shown in Figure 7 and Figure 8 respectively. For the 11 gene network, MDL inferred a total of 19 edges of which seven were correct where as TLMI based network MDL approach inferred a total of 17 edges of which eight were correct, resulting in an improvement in both precision and recall. For the 9 gene network, the network MDL approach inferred a total of nine edges of which three were correct where as the proposed TLMI based MDL approach inferred a total of six edges of which three were correct. In this case, while recall for both methods is the same, the precision of the proposed approach is better.

### TLMI and TLCMI based PMDL Implementation

We also incorporated the proposed TLMI and TLCMI metrics in the PMDL based algorithm. A network with 14 genes was derived from the yeast cell cycle [26-28] and Spellman’s data [25] was used again for performance evaluation. The new PMDL implementation inferred 27 edges of which five were correct while the earlier PMDL algorithm inferred 26 edges of which five were correct (the true and inferred biological networks from this phase have not been shown). While the numbers are close the correctly inferred edges were different. The comparable performance of the two PMDL implementations point to a need for further investigation on the time-lagged CMI metric. The precision and recall values for the algorithms are given in Table 2.

**Table 2:** Performance of MI and TLMI based PMDL

Method Metric	PMDL	Time Lagged PMDL
Precision	22.73%	19.23%
Recall	22.73%	18.52%

### Performance: Time and Space complexities of proposed algorithms

**Figure 9:**
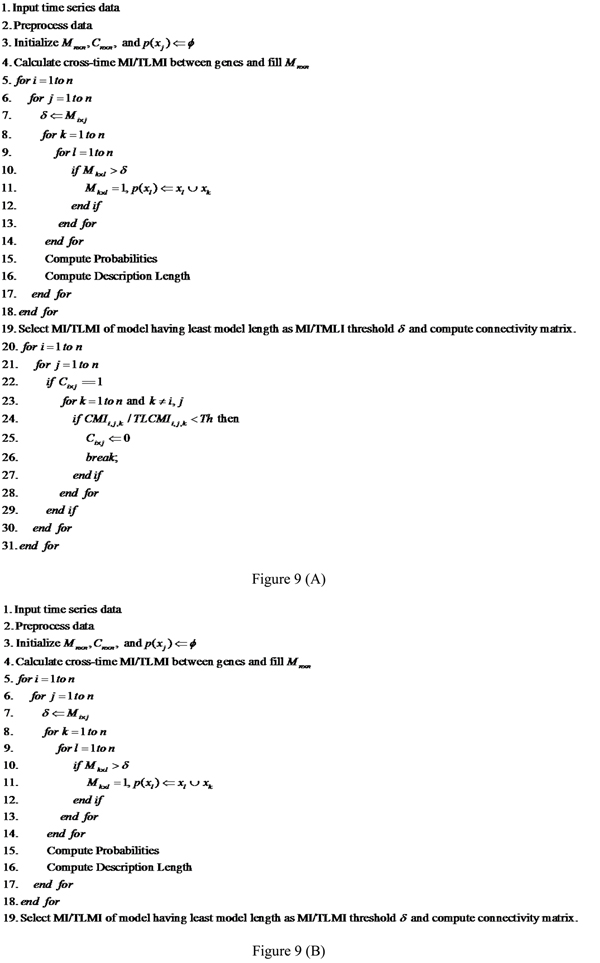
**TLMI and MI based network MDL and PMDL algorithms.** Figure 9(a) – TLMI and MI based PMDL algorithm. Figure 9(b) – TLMI and MI based network MDL algorithm.

The performance of the PMDL algorithm depends on three factors: the number of genes, the number of time points and most importantly the number of parents inferred for each gene by the algorithm. To see what role these factors play we will look into the time and space complexities of the algorithm. A schematic for the PMDL algorithm is shown in Figure 9(A) while that for the Network MDL algorithm is shown in Figure 9(B).

Step 4 of the PMDL algorithm iterates *n*^2^(*m* − *τ*) times where *n* is number of genes, *m* is the number of time points, and *τ* is the time lag. From line 5 to line 18 the algorithm iterates *n*^4^ times, lines 15 and 16 of the algorithm iterates *n*^3^(*m* − *τ*) times. Finally from lines 20 to 31 the algorithm iterates *n*^3^ times. Thus the time complexity of the over-all algorithm is Θ(*n*^4^ + *n*^3^(*m* − *τ*)). 

From the time complexity it can be seen that if the number of genes is larger than the number of time points then the run time of PMDL algorithm depends on the number of genes. And if the number of time points is larger than the number of genes then the run time depends on the number of time points. In the worst case  is zero for all genes and the algorithm runs in Θ(*n*^4^ + *n*^3^*m*) time.

When it comes to space complexity, the conditional probability tables play a major role. If a gene has *n* parents then the conditional probability tables take 2*^n^* units of space. Thus, the amount of memory needed by the algorithm depends on the number of parents inferred by the network. As the space complexity grows exponentially based on the number of parents it is possible that the algorithm may run out of memory for a data set with as few as 50 genes whereas it may run for as little as 5 minutes for a data set with several hundred genes. There are 2 ways to overcome this limitation: 

1. Restrict the number of parents.

2. Take the next smallest description length, instead of using the smallest one.

The first approach will guarantee results when the number of parents is restricted to a small value but this may lower the accuracy of the result. The second approach may take more time to run but as we are not restricting the number of parents the accuracy of the algorithm is not affected. Some bench marking studies are required on the above two approaches to see which one works best.

In the MDL based implementation we discard the lines 20 to 31 from the PMDL based implementation. The worst case time complexity is again Θ(*n*^4^ + *n*^3^*m*).

## Conclusions

In this paper, we have studied the effects of cDNA microarray data size on three algorithms: PMDL, network MDL, and a DBN based approach. The study shows that the data size plays an important role in the inference accuracy of each of these algorithms. The experiments were carried out on synthetically generated time-series data and the performance saturation points were listed for these algorithms. The immediate benefit of this work lies in helping biologists to devise cDNA microarray experiments intelligently depending on the class of GRN inference algorithms they are likely to use to achieve maximum accuracy. In a bid to understand the performance saturation of the information theoretic approaches, we also found out that mutual information saturates and effectively tends to zero as the entropy in the network increases with increase in the data size. These observations lead us to believe that MI by itself might not be the best metric in devising information theoretic approaches for GRN inference. The DBN approach however showed good performance only for a smaller data size which is non-intuitive and requires further analysis for validation. Based on these findings, we introduced two new information theory metrics viz. TLMI and TLCMI and used them in the network MDL and PMDL based algorithms to develop two novel GRN inference algorithms. The results indicate that transcriptional time lags play an important role in gene regulatory network inference methods as evidenced by the higher accuracy provided by our algorithm.

## Methods

### Time Lags

The concept of time lags was first introduced by Zou [18], where they proposed that the time difference between the initial expression change of a potential regulator (parent) and its target gene represents a biologically relevant time period. Here potential regulators are those set of genes whose initial expression change happened before the target gene. Also initial expression change is up or down-regulation (ON or OFF) of genes. 

**Figure 10:**
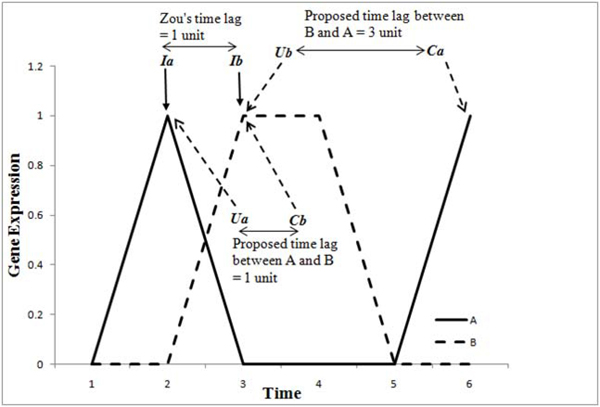
Time lag computation schemes

Our motivation lies in changing the MI metric to incorporate the effect of time lags. While implementing Zou’s method of calculating time lags, we come across the problem of negative lags. For every pair of gene when the initial expression change is not at the same time point, one of the two time lags turns out to be negative. Figure 10 illustrates this problem. In the figure ***Ia*** and ***Ib*** indicate the ***initial change in expression*** of gene A and gene B at time points 2 and 3 respectively. As per Zou [18], gene A is parent of gene B and they do not consider the time lags between B and A. Time lag between A and B is ***Ia-Ib*** = 3-2 = 1. Time lag between B and A is ***Ib-Ia*** = 2-3 = -1. This gives a negative time lag while we try to build the MI-matrix for every possible pair of genes. It is important to consider such time lags both in the forward and backward directions (i.e., from A to B and vice versa) as this can model the loops between 2 genes (i.e., A->B and B->A connections).

In biological networks the A↔ B schema is quiet common. Hence Zou’s  time lag computation scheme needs to change to handle such cases. We also argue that a gene can affect another gene only when it is up-regulated (ON). Based on the above discussion, ***we propose time lags as the difference between initial up-regulation of first gene and initial expression change of the second gene after the up-regulation of first gene.*** This solves the issue of negative time lags besides being biologically more relevant as compared to the existing method of calculating time lags. Figure 10 also illustrates the proposed time lag computation based on our approach. In the figure, ***Ua*** and ***Ub*** indicate the ***initial up-regulation*** of genes A and B at time points two and three respectively. ***Ca*** and ***Cb*** indicate that time points six and three are the time points where the expression values of genes A and B ***changed after the initial up-regulation*** of genes B and A. Time lag between A and B is calculated as **τ1 = *Cb*-*Ua*** and time lag between B and A is calculated as **τ2 = *Ca*-*Ub*** respectively. In this example time lag between X and Y is one and time lag between Y and X is three.

### Information Theoretic metrics

#### *Entropy, joint entropy, mutual information and conditional mutual information*

*Entropy, H*, is the measure of the average uncertainty in a random variable [19]. If *p_i_* is the probability of observing a particular symbol in a sequence then entropy is given as 

As the proposed algorithm quantizes the microarray data to two levels, a gene takes two values a 0 and 1 corresponding to being in OFF and ON states respectively. In this case, the entropy of a gene *A* is defined as  , where *p_0_* and *p_1_* are the probabilities of observing a gene A as 0 and 1 respectively over the sequence *A *[15]. Here sequence *A* contains the values taken by a gene in the time series data; thus if we have a time series data of *m* time points then sequence A is of length *m*.

This standard definition of entropy has been used in the MDL and PMDL schemes where the entropy was computed for sequence length of *m-1* to simulate a default time-lag of 1, i.e., compute the entropy between A (considering its expression values from 1, 2,..., m-1)  and B (considering its expression values from 2, 3,..., m). In order to implement the TLMI and TLCMI metrics, we need to compute the entropies between A (considering its expression values from 1, 2,..., m-τ) and B (considering its expression values from τ+1, τ+2,..., m).

*Joint Entropy* between two sequences *A* and *B* , *H*(*A, B*) is defined as 

Thus joint entropy between two variables is an extension of entropy where the two sequences (*A*, *B*) are considered to be a single vector valued random variable dependent on each other [19].

As stated before, the proposed algorithm quantizes the microarray data into two levels, in this case the joint entropy between two sequences *A* and *BH*(*A, B*) is defined as  where *p*_0,0_, *p*_0,1_, *p*_1,1_ and *p*_1,1_ are the probabilities of observing both zeros, a zero and a one, a one and a zero and both ones in sequences *A* and *B* respectively.

*Mutual Information* measures the amount of information that can be obtained about one random variable by observing another one [19]. 

MI in terms of entropies is defined as [19] and the classical MI metric is symmetric i.e. 

*Conditional Mutual Information* is the reduction in the uncertainty of A due to knowledge of B when C is given [19]. High MI indicates that there may be a direct or indirect relationship between the genes [14]. To overcome this issue, Zhou [3] implemented the concept of CMI. CMI in terms of entropy is defined as  . We have seen the computation of entropy and joint entropy of two variables before. The CMI involves computation of joint entropy between 3 variables which can be extended based on the joint entropy between two variables. Again as the proposed algorithm quantizes the microarray data to two levels, the joint entropy between three sequences *A*, *B* and *C*, *H*(*A*,* B*,* C*) is defined as

#### *Time lagged mutual information (TLMI) and time lagged conditional mutual information (TLCMI)*

After implementing time lags, we no longer compute the entropy and joint entropy over the complete sequences of gene(s) as discussed before. If a time lag τ is computed between two genes *A* and *B* we remove the last τ symbols of sequence *A* and first τ symbols of sequence *B* to obtain reduced sequences (of length m-τ each) for *A* and *B* respectively. Computing the MI over these reduced sequences gives TLMI.

TLMI is not a symmetric quantity though, i.e.  .

Considering a time lag, τ, between A and B, we compute TLCMI(A;B|C) by deleting the last τ symbols in sequences A and C (i.e., look into the sequences from time points 1, 2,..., m-τ) and first τ symbols in sequence of B (i.e., look into the sequences from time points τ+1, τ+2,..., m) and computing the CMI of these reduced sequences to obtain TLCMI.

**Figure 11:**
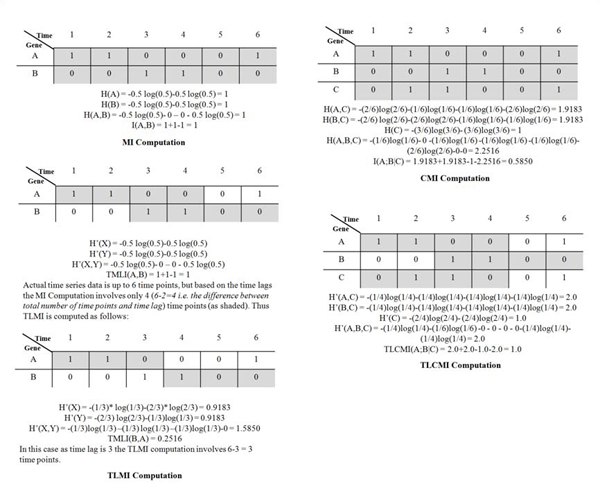
Classic and proposed information theory metrics

Figure 11 demonstrates computation of the information theoretic quantities discussed above.

### Minimum Description Length (MDL) and Predictive Minimum Description Length (PMDL) Principle

Estimating the MI threshold is one of the major drawbacks in information theory based model. Zhao . [12] first proposed the MDL principle to solve the problem. Based on the microarray data, the MI matrix is computed. If the microarray data has *n* genes then two n*n matrices viz. one connectivity matrix and one MI matrix are stored. A time lag of one unit is assumed, thus the MI computations are not symmetric. Using every MI value as a threshold over the MI matrix, n^2^ models are obtained. For every model, the description lengths (model length + data length) are computed and the model with the minimum description length is selected as the best model. This algorithm involved a fine tuning parameter for the model length algorithm which also makes the MDL principle method arbitrary [16]. To overcome this issue we earlier proposed a PMDL based inference algorithm [17]. In the PMDL principle, we discard the model length while computing the data length of each model. The results increased both true edges and false edges in inferred networks. In order to reduce the false edges, CMI was applied over the best model and hence the over-all PMDL based approach improved the inference accuracy over the conventional network MDL scheme [17].

### Time lagged based MDL and PMDL implementation

The basic information theoretic metrics with unit time lag were replaced in the network MDL and PMDL algorithms with the TLMI and TLCMI metrics. Figure 9 illustrates the existing and proposed algorithms. Figure 9A shows the MI and TLMI based PMDL algorithm. While both the existing and our proposed algorithms are shown in the same figure, it is to be noted that the difference lies in using the right information theoretic metrics (lines 4, 19 and 24 in Figure 9A). Figure 9B shows the MI and TLMI based network MDL algorithms.  Further explanations on the PMDL and network MDL algorithms can be found in [17] and [12] respectively.

## Competing interests

The authors declare that they have no competing interests.

## Authors' contributions

VC, CZ and PG1 developed the algorithm and implemented the algorithm on synthetic and biological data sets. An in-depth analysis of results was also performed on the results. EJP and PG2 coordinated the study.
